# Multiparametric Monitoring of Disease Progression in Contemporary Patients with Wild-Type Transthyretin Amyloid Cardiomyopathy Initiating Tafamidis Treatment

**DOI:** 10.3390/jcm13010284

**Published:** 2024-01-04

**Authors:** Svenja Ney, Roman Johannes Gertz, Lenhard Pennig, Richard J. Nies, Udo Holtick, Linus A. Völker, Gilbert Wunderlich, Katharina Seuthe, Christopher Hohmann, Clemens Metze, Claas Philip Nähle, Jennifer von Stein, Monique Brüwer, Henrik ten Freyhaus, Roman Pfister

**Affiliations:** 1 Department III of Internal Medicine, Faculty of Medicine and University Hospital Cologne, University of Cologne, 50937 Cologne, Germany; richard.nies@uk-koeln.de (R.J.N.); katharina.seuthe@uk-koeln.de (K.S.); christopher.hohmann@uk-koeln.de (C.H.); clemens.metze@uk-koeln.de (C.M.); jennifer.von-stein@uk-koeln.de (J.v.S.); monique.bruewer@uk-koeln.de (M.B.); henrik.ten-freyhaus@uk-koeln.de (H.t.F.); roman.pfister@uk-koeln.de (R.P.); 2 Department of Radiology, Faculty of Medicine and University Hospital Cologne, University of Cologne, 50937 Cologne, Germany; roman.gertz@uk-koeln.de (R.J.G.); lenhard.pennig@uk-koeln.de (L.P.); cp@naehle.net (C.P.N.); 3 Department I of Internal Medicine, Faculty of Medicine and University Hospital Cologne, University of Cologne, 50937 Cologne, Germany; udo.holtick@uk-koeln.de; 4 Department II of Internal Medicine and Center for Molecular Medicine Cologne, Faculty of Medicine and University Hospital Cologne, University of Cologne, 50937 Cologne, Germany; linus.voelker@uk-koeln.de; 5 Cologne Cluster of Excellence on Cellular Stress Responses in Ageing-Associated Diseases, 50923 Cologne, Germany; 6 Department of Neurology and Center for Rare Diseases, Faculty of Medicine and University Hospital Cologne, University of Cologne, 50937 Cologne, Germany; gilbert.wunderlich@uk-koeln.de; 7 Radiologische Allianz Hamburg, 20357 Hamburg, Germany

**Keywords:** amyloidosis, transthyretin amyloid cardiomyopathy, cardiac magnetic resonance imaging, tafamidis

## Abstract

Background: Recently, a disease modifying therapy has become available for transthyretin amyloid cardiomyopathy (ATTR-CM). A validated monitoring concept of treatment is lacking, but a current expert consensus recommends three clinical domains (clinical, biomarker and ECG/imaging) assessed by several measurable features to define disease progression. Methods: We retrospectively analyzed data of wild-type ATTR-CM patients initiating tafamidis therapy assessed within our local routine protocol at baseline and 6-months follow-up with respect to the frequency of values beyond the proposed thresholds defining disease progression. Additionally, associations of cardiac magnetic resonance (CMR) tomography with clinical domains were examined within a subgroup. Results: Sixty-two ATTR-CM patients were included (88.7% male, mean age 79 years). In total, 16.1% of patients had progress in the clinical and functional domain, 33.9% in the biomarker domain and 43.5% in the imaging/electrocardiography (ECG) domain, with the latter driven by deterioration of the diastolic dysfunction grade and global longitudinal strain. In total, 35.5% of patients showed progress in none, 35.5% in one, 29.0% in two and no patient in three domains, the latter indicating overall disease progression. A subgroup analysis of twenty-two patients with available baseline and follow-up CMR data revealed an increase in CMR-based extracellular volume by more than 5% in 18.2% of patients, with no significant correlation with progress in one of the clinical domains. Conclusions: We provide first frequency estimates of the markers of disease progression according to a recent expert consensus statement, which might help refine the multiparametric monitoring concept in patients with ATTR-CM.

## 1. Introduction

Transthyretin amyloid cardiomyopathy (ATTR-CM) is a progressive and potentially fatal disease caused by the accumulation and extracellular deposition of transthyretin-derived insoluble amyloid fibrils in the myocardium [1]. It was generally regarded an orphan disease with adverse prognosis, but recent epidemiological data showed an increasing prevalence of cardiac amyloidosis during the last ten years which mainly is driven by wild-type ATTR-CM [2,3]. In 2020, tafamidis was the first therapy to be approved for the treatment of wild-type- and hereditary ATTR-CM, which changed the disease course from incurable to treatable. In the pivotal “Transthyretin Amyloidosis Cardiomyopathy Clinical Trial (ATTR-ACT)”, tafamidis treatment compared to placebo was shown to significantly improve mortality, morbidity, and functionality in patients with ATTR-CM [4].

Consistent with the pharmacological effect of tafamidis, a stabilization and attenuation of the disease progression was observed rather than a reversal or cure of the disease. Given that the first patients have now been treated with tafamidis for a few years, the important question of how to monitor therapy and disease progression arises. Disease progression monitoring is quintessential to identify non-responders particularly in the context of pronounced treatment costs. Furthermore, new and even more effective therapies are currently under evaluation and may become available in the near future for therapy escalation (HELIOS-B: A Study to Evaluate Vutrisiran in Patients with Transthyretin Amyloidosis With Cardiomyopathy; NCT04153149; ION-682884 in Patients With TTR Amyloid Cardiomyopathy; NCT04843020). Garcia-Pavia et al. [5] recently published an expert consensus for monitoring the course of patients with ATTR-CM. Assessments every 3 to 6 months with evaluation of three separate domains—clinical and functional endpoints, laboratory biomarkers, and imaging and electrocardiographic parameters—were proposed with meaningful thresholds of respective measurable markers defining deterioration. Disease progression should be assumed, if at least one marker from each of the three domains deteriorates. However, clinical experience with this strategy is lacking. In particular, the frequency and degree of deterioration within the respective domains remains elusive. Furthermore, cardiovascular magnetic resonance (CMR) derived T1-mapping and extracellular volume (ECV) facilitate the non-invasive assessment of intra- and extra-cellular myocardial compartments separately and is considered a surrogate for amyloid burden [6,7]. To date, the added value of T1-mapping and ECV to improve the measurement of disease progression is still uncertain.

In this pilot study, we analyzed the frequency of disease progression in a contemporary cohort of 62 patients with ATTR-CM during the first 6 months after initiation of tafamidis therapy by assessing several measurable features of the three recently suggested domains of disease manifestation, and additionally, by quantifying amyloid load by CMR within a subgroup of 22 patients.

## 2. Methods

### 2.1. Setting and Study Design

This study was approved by the local institutional review board (Ethics committee of the Faculty of Medicine from the University of Cologne, Cologne, Germany). Necessity for informed consent was waived due to the retrospective design of the study. All clinical investigations were conducted in accordance with the Declaration of Helsinki.

In this retrospective study, all patients with TTR amyloidosis and cardiomyopathy who were treated at the amyloidosis center of the University Hospital of Cologne and initiated therapy with tafamidis 61 mg o.d. after approval in Germany between April 2020 and December 2021 were deemed eligible. Only patients with available baseline and follow-up CMR data were considered for CMR analysis. All data were retrospectively collected from medical records and analyzed from an anonymized dataset.

### 2.2. Diagnostic Approach for ATTR-CM and Clinical Assessment

At our institution, patients undergo a standardized diagnostic workup for suspected cardiac amyloidosis. Cardiac TTR amyloidosis is diagnosed in accordance with current guidelines either noninvasively through bone scintigraphy Perugini II or III in absence of monoclonal gammopathy in serum and urine immunofixation and a normal free light chain assay [8] or—in case of inconsistent or abnormal non-invasive test results—through endomyocardial biopsy and subsequent immunohistochemical classification of amyloid [9]. Sequencing of the TTR gene was recommended to all patients. Every case was discussed in a multidisciplinary amyloidosis board to determine the diagnostic and therapeutic strategy.

Before the initiation of treatment with tafamidis and after 6 months of therapy, patients underwent a standardized clinical and laboratory assessment as part of routine care, including New York Heart Association (NYHA) functional class, Minnesota Living with Heart Failure Questionnaire (MLHFQ) and cardiac biomarkers (NT-pro BNP, Troponin T), hemoglobin- and glomerular filtration rate (GFR) levels as well as an instrumental assessment (resting ECG, transthoracic echocardiography and CMR). Transthoracic echocardiography was performed at our echocardiography lab (GE Vivid E 95). Left-ventricular (LV) dimensions, LV wall thickness, LV ejection fraction (LVEF) (definition of LVEF: preserved EF: ≥50%; mid-range EF 41–49%; reduced EF ≤ 40%), stroke volume and global longitudinal strain (GLS) were analyzed according to the recommendations for cardiac chamber quantification by echocardiography in adults (American Society of Echocardiography and the European Association of Cardiovascular Imaging) [10]. Diastolic function was assessed according to the recommendations for the evaluation of left ventricular diastolic function by echocardiography (American Society of Echocardiography and the European Association of Cardiovascular Imaging) [11]. Image analyses were performed through Tomtec, Image-Arena Version 4.6 by one experienced member of the study group to reduce interobserver variability.

### 2.3. Definition of Disease Progression in TTR-CM

Disease progression was defined according to the recently published expert consensus on monitoring of ATTR-CM using three separate domains: clinical/functional, laboratory biomarker, and imaging/ECG. At least one marker from each of the three domains was required to determine disease progression [5]. To evaluate disease progression in the first domain—clinical and functional criteria—we assessed heart failure related hospitalizations, increases in the NYHA functional class, and increases in the Minnesota Living with Heart Failure Questionnaire (MLHFQ ≥ 10% from baseline). As recommended, all NYHA measures were captured after a 30-day period of stabile symptoms.

To evaluate disease progression in the second domain—laboratory biomarker—we analyzed the increase in high sensitivity troponin T (≥30% from baseline; Elecsys Troponin T hs STAT, Roche Diagnostics, Indianapolis, IN, USA) and NT-pro BNP levels (relative increase of ≥30% from baseline or absolute increase of 300 pg/mL; Elecsys proBNP II, Roche Diagnostics) as well as advances in the National Amyloidosis Center (NAC) staging scale [12]. NT-pro BNP levels were taken at steady state with no change in symptoms or need for adjustment of the administered dose of diuretics over the past 30 days.

To evaluate disease progression in the third domain—imaging and ECG—we assessed the new onset of conduction disturbance in ECG and a range of echocardiography parameters such as increases in LV wall thickness (≥2 mm), increases in diastolic dysfunction grade or changes in systolic measurement (≥5% decrease in LVEF; ≥5 mL decrease in stroke volume, ≥1% increase in GLS).

For CMR, significant progression in ECV fraction was defined as an absolute increase ≥ 5% compared to baseline [13].

### 2.4. Cardiac Magnetic Resonance

CMR imaging was performed using a commercially available whole body 1.5 T MRI system (Philips Ingenia, Philips Healthcare, Best, The Netherlands) equipped with a 28-channel coil for cardiac imaging. The standardized imaging protocol comprised 2D balanced steady-state free precession cine sequences in standard orientations (short axis [SA], 4-chamber [4Ch], 2-chamber [2Ch], and 3-chamber [3Ch]). T2-weighted fat-saturated black blood images were acquired in SA. Prior to admission of gadolinium contrast, T1-mapping (using a 3(3)3(3)5 modified look-locker inversion recovery sequence [14]) and T2-mapping (using a gradient-spin-echo sequence [15]) were acquired in SA (3 slices; apical, mid-ventricular, and basal). Afterwards, Gadobutrol (Gadovist, Bayer HealthCare Pharmaceuticals, Berlin, Germany; 0.2 mmol/kg) was injected into an antecubital vein (flowrate of 2 mL/s). 10 min after injection of gadolinium contrast, T1-mapping images were acquired in SA (3 slices). T1-weighted inversion-recovery fast spoiled gradient-echo sequences were used in standard orientations (SA, 4Ch, 2Ch, and 3Ch) for late gadolinium enhancement (LGE) imaging. All sequences were acquired during end-expiratory breath-hold in end-diastole.

Two radiologists with 3 (RJG) and 6 (LP) years of experience in CMR analyzed the data and performed the measurements in consensus using commercially available software (IntelliSpace Portal version 10.1; Philips Healthcare, Best, The Netherlands). Readers were blinded to the clinical information. In order to minimize potential recall bias, there was an interval of four weeks between analysis of baseline and follow-up CMR. All volumes and masses were indexed to the body surface area at the day of the examination employing the Mosteller [16] method. Myocardial T1-relaxation times and ECV fraction values (using pre- and post-contrast T1 values) were calculated using a segmental approach as described previously [17].

### 2.5. Statistics

Variables are described and presented as frequencies and percentages, mean and standard deviation (SD) or median and interquartile range (IQR), as appropriate. All variables relevant for further analyses were examined for normal distribution (Shapiro–Wilk test).

Differences between baseline and the 6-months follow-up were analyzed using dependent samples t-test for normally distributed variables, and Wilcoxon signed-rank test. All reported *p*-values are two-sided and *p*-values < 0.05 were considered statistically significant. Statistical analyses were performed using IBM SPPS Statistics, Version 28.

## 3. Results

### 3.1. Baseline Characteristics and Comorbidities

During the course of the study, 82 patients were diagnosed with ATTR-CM at our institution. 62 of these initiated specific therapy with tafamidis had a complete follow-up and were included in the study. Of these patients, 22 underwent baseline- and follow-up CMR at 6 months while the remaining patients did not undergo CMR assessment due to previous or subsequent pacemaker-implantation, lack of consent or nonattendance at the baseline or follow-up.

Details of baseline demographic parameters and comorbidities are shown in Table 1. Mean age of this predominantly male (88.7%) cohort was 79.0 (SD 6.4) years. Genetic testing was performed in 47 of 62 patients (75.8%). The remaining 15 patients refused genetic testing, with 11 of them being over 80 years old at the time of diagnosis. The most common comorbidity among the ATTR-CM patients was arterial hypertension, which was present in 74.2%, followed by atrial fibrillation (59.7%). In total, 40.3% of the patients suffered from coronary artery disease, and 9.7% experienced previous cerebrovascular events. In more than half (54.8%) of the patients, polyneuropathy was present, 45.2% of patients suffered from carpal tunnel syndrome, and seven patients (11.3%) were previously diagnosed with spinal canal stenosis. At baseline, 88.7% of the patients were symptomatic with NYHA functional class II or III. A total of 58.1% of the patients were treated with loop diuretics, with a median dose of 20 mg furosemide per day (IQR 10–37.5). At baseline more than half (57.4%) of the patients were in NAC stage I, 29.5% were in stage II, and 13.1% were in stage III.

### 3.2. Course of Clinical and Functional Parameters

Details on clinical parameters, as well as laboratory, echocardiographic, and CMR data at baseline- and 6-months follow-up are shown in Table 2. There were no decompensation-related hospitalizations requiring intravenous diuretic treatment. At the 6-months follow-up, 74.2% of the patients had treatment with loop diuretics, with a median dose of 20 mg furosemide per day (IQR 10–40). Compared to baseline, loop diuretics were either newly initiated or slightly increased in 27.4% of the patients. In 6.5% of the patients, dose was reduced. NHYA functional class was stable in 74.2% of patients, while 12 patients (19.4%) reported an improvement in NYHA functional class and only four patients (6.5%) experienced worsening of symptoms. Six patients experienced worsening of Quality of Life, defined as a MLHFQ increase ≥ 10%, while the remaining patients experienced stabilization or improvement in the MLHFQ.

**Figure 1:**
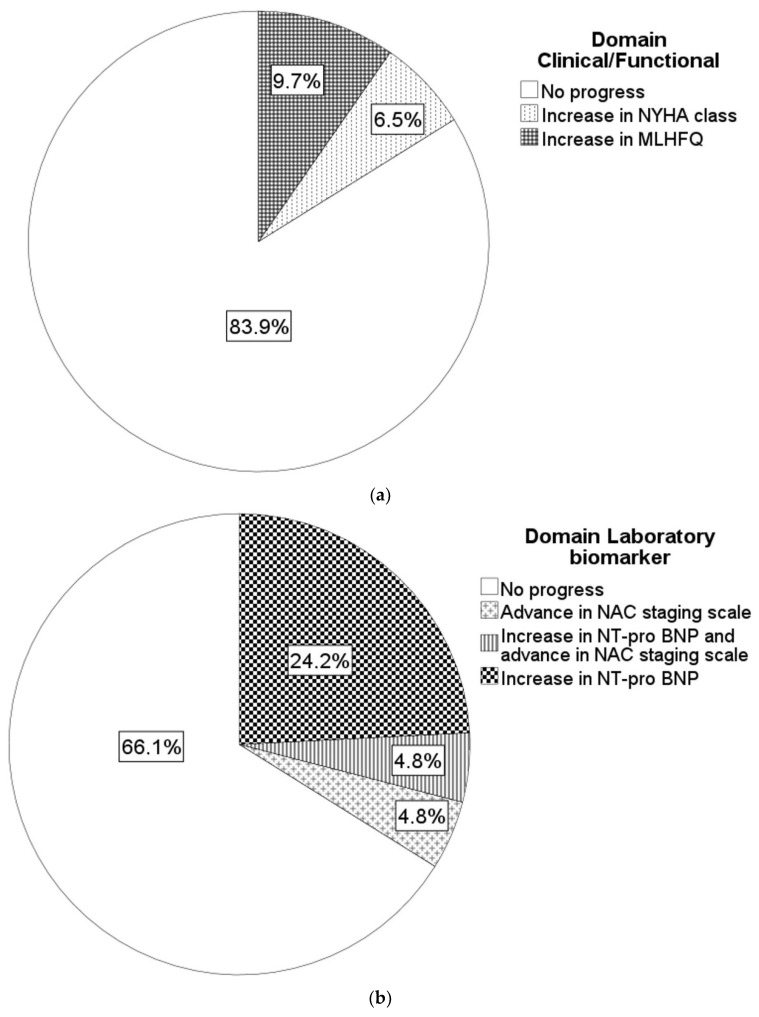

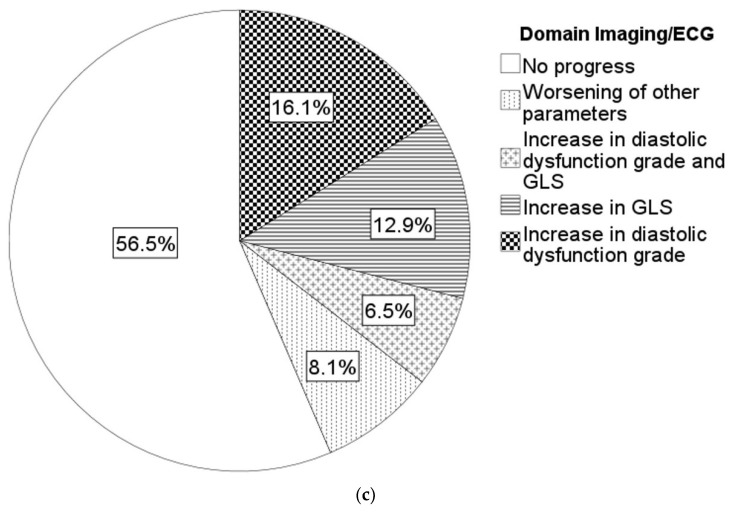
(**a**–**c**): Individual markers and types of domains with progression. (**a**) Domain Clinical/Functional; (**b**) Domain Laboratory biomarker; and (**c**) Domain Imaging/ECG.

Overall, ten (16.1%) patients met the criteria for progression in the domain “clinical and functional criteria”, mainly driven by the worsening of Quality of Life (Figure 1a).

### 3.3. Biomarkers and Laboratory Parameters

Cardiac biomarkers such as NT-pro BNP and Troponin T were significantly elevated in ATTR-CM patients with a median serum troponin T of 0.053 µg/L (IQR 0.036–0.088) and a median serum NT-pro BNP of 2,177 ng/L (IQR 1,271–3,848) at baseline. Renal function was preserved with a median GFR of 59 mL/min (IQR 47–74). At the 6-months follow-up, none of the patients showed significant increase in serum troponin T levels, while 18 patients (31.0%) revealed significant increase in serum NT-pro BNP levels. Six (10.3%) patients showed worsening of the NAC stage, while 10.3% showed improvement of the NAC stage and the remaining 79.3% revealed no change in the NAC stage at the 6-months follow-up compared to the baseline.

Overall, 21 (33.9%) patients met the criteria for progression in the domain “laboratory biomarkers”, mainly caused by increase in NT-pro BNP levels (Figure 1b).

### 3.4. Echocardiography and ECG

LV thickening was present in all patients with a median interventricular septum thickness of 17 mm (IQR 16–18) at baseline. One patient experienced disease progression in terms of increase in LV wall thickness ≥ 2 mm at the 6-months follow-up. Diastolic dysfunction was present in all patients in this cohort. At baseline, almost two thirds (57.4%) of the patients showed diastolic dysfunction ≥ grade II, while at the 6-months follow up diastolic dysfunction ≥ grade II was present in 62.9%. Sixteen (25.8%) patients experienced worsening of the diastolic dysfunction grade. Analysis of systolic LV function revealed a preserved LVEF in 82.3% of patients at baseline. Eight patients showed mid-range LVEF, and three patients presented with reduced LVEF. Stroke volume index was slightly impaired at baseline (median 31 mL/m^2^, IQR 26–39). Analysis of longitudinal LV function revealed a significantly reduced GLS (median −11%; IQR −8.5 to −14.2).

One patient experienced worsening in LVEF (midrange to reduced), while one patient showed improvement from reduced to mid-range LVEF. Stroke volume index showed significant improvement during the 6 months course (31 vs. 34 mL/m^2^, *p* < 0.01). Worsening in GLS occurred in fourteen (22.6%) patients.

Four patients experienced a new onset of conduction disturbance (new onset of AV block/PR interval prolongation > 20 ms) on resting ECG, with one patient requiring pacemaker implantation.

Overall, 27 patients (43.5%) met the criteria for progression in the domain “Imaging and ECG”, mainly driven by worsening of GLS and diastolic dysfunction (Figure 1c).

### 3.5. Overall Disease-Progression

**Figure 2:**
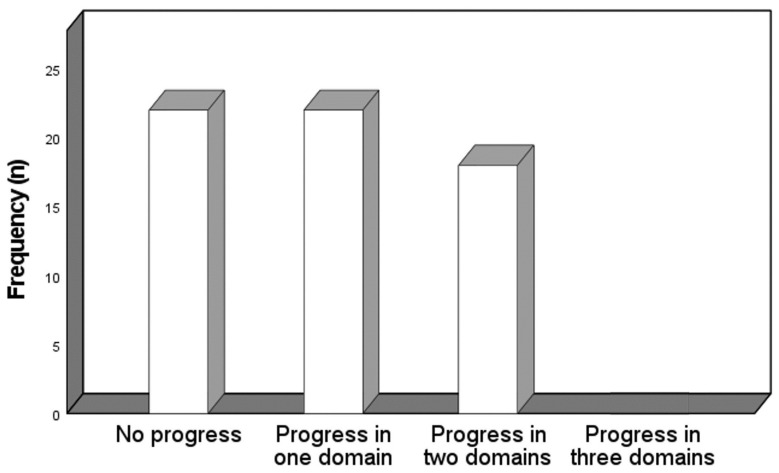
Frequency of domains with progression.

Parameters of disease progression were met in either none (35.5%), one (35.5%) or two (29.0%) of the above-mentioned domains. There was no patient with overall disease progression, defined as worsening of at least one marker from each of the three domains (Figure 2). The majority of patients with progress in one domain progressed in the domain imaging/ECG (17.7%), followed by the laboratory biomarker (9.7%) and the clinical/functional (8.1%) domain. The patients with two progressive domains comprised imaging/ECG and laboratory biomarker (21.0%), imaging/ECG and clinical/functional (4.8%) and clinical/functional and laboratory biomarker (3.2%).

### 3.6. CMR Parameters

**Figure 3:**
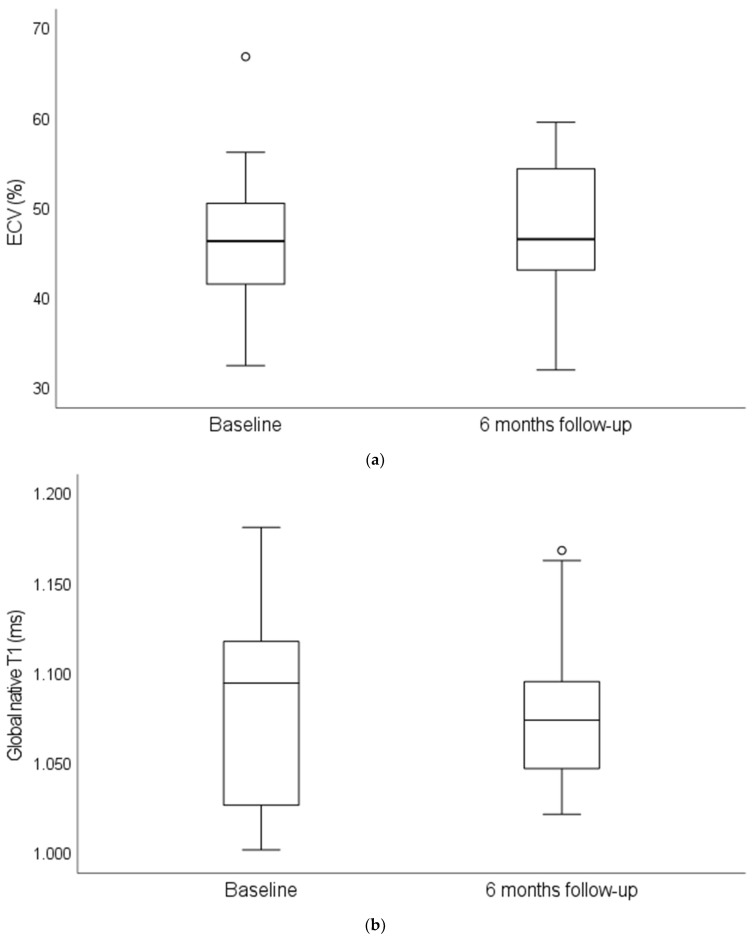
(**a**,**b**) Parameters of cardiac magnetic resonance imaging reflecting amyloid load at baseline and 6-months follow-up (*n* = 22 patients). (**a**) ECV (%) at baseline and 6-months follow-up; (**b**) Global native T1 (ms) at baseline and 6-months follow-up.

Compared to in-house reference values for T1-relaxation times (961–985 ms) and ECV fraction (23–28%), both parameters were significantly elevated at baseline (T1:1094.8 ms (IQR 1002.0–1671.1); ECV: 46.4% (IQR 32.5–66.9)). For both parameters, no significant change was observed during follow-up (T1:1047.2 ms (IQR 969.2–1168.6); ECV: 46.6% (IQR 32.0–59.6)) (Figure 3). Four patients showed an absolute increase in ECV fraction ≥ 5% within our study cohort.

Among these four patients, two experienced progress in one other domain (imaging/ECG), while one patient showed progress in two domains (imaging/ECG and laboratory biomarker). The rate of patients with progressive domains did not differ significantly between patients with increase in ECV fraction and patients without increase in ECV fraction.

## 4. Discussion

In this cohort of patients with ATTR-CM initiating tafamidis therapy, deteriorated parameters at the 6-months follow-up proposed for defining disease progression were met in 35.5% of patients in none, in 35.5% of patients in one and in 29.0% of patients in two of the three domains clinical/functional, laboratory biomarker, and imaging/ECG. No patient had deteriorated parameters in all three domains revealing the proposed definition of overall disease progression. Deteriorated grade of diastolic dysfunction and GLS were found most frequently indicating progression of the domain imaging/ECG in almost half of the patients. Importantly, however, mean values of the examined markers showed no significant deterioration within 6 months of treatment with tafamidis. ECV assessed in CMR showed evidence for progression in 18% of patients with no significant association with the three clinical domains.

Albeit 24% of hereditary ATTR cases were included and only outcomes for the total cohort were reported, the ATTR-ACT trial generated relevant data on serial changes of disease markers in patients with wild-type ATTR-CM on tafamidis therapy, which comprises the vast majority of patients found in routine care. With respect to the parameters examined in our study and proposed by the current expert consensus on disease monitoring, the rate of cardiovascular hospitalization in ATTR-ACT was 0.55 per year in patients treated with tafamidis. Despite significantly less pronounced than in the placebo group, mean values of markers of functionality, quality of life, and cardiac function such as wall thickness, GLS, and stroke volume index declined during the trial period indicating disease progression despite tafamidis treatment. So far, the rate of patients with deteriorating disease markers indicating relevant disease progression have not been reported for the trial population [4].

Since we are—to the best of our knowledge—the first group evaluating disease progression according to the recent expert consensus [5], comparison with other studies is somewhat limited. Generally, real-world and systematic data apart from the approval trial on the clinical course of contemporary patients with tafamidis treatment are sparse. Considering one quarter of patients with hereditary ATTR in ATTR-ACT, the baseline characteristics in our cohort are comparable with the ATTR-CM population and other available real-world populations [18,19,20]. Chamling et al. [19] analyzed 20 patients in a monocentric study with wild-type ATTR-CM treated with tafamidis and reported changes of selected parameters at 12-months follow-up. NYHA class did not change significantly but median NT-pro-BNP levels showed a significant increase of about 300 pg/mL indicating a substantial part of patients fulfilling the progression threshold in the biomarker domain. Rettl et al. [18] analyzed 35 patients in a monocentric study with wild-type ATTR-CM treated with tafamidis in a compassionate use program before formal approval. A significant improvement of NYHA class was observed whereas the mean levels of the 6-min walking distance and the biomarkers troponin and NT-pro-BNP did not change significantly at 9-months follow-up. A dedicated analysis on the frequency of deteriorated markers was not provided in both studies. A small monocentric study from Japan analyzed 18 patients with ATTR-CM and tafamidis treatment, of whom 94% had the wild-type disease [21]. In five patients, an increase in troponin I was detected, which was associated with clinical outcome. Lastly, a study on 101 patients with ATTR-CM and 84% wild-type disease reported no relevant changes of median NT-pro-BNP and troponin levels during a mean of 8.5 months of tafamidis treatment but an increase in median septal thickness of 2 mm meaning that half of the population has a progression within the proposed imaging/ECG domain [22]. Taken together, despite some preliminary experience with tafamidis, so far, no study focused systematically on prevalence of disease progression and associated quantitative markers.

Progression of the clinical/functional domain in our cohort was driven by a quality of life questionnaire. No hospitalizations occurred which is not unexpected considering the 6-months follow-up and the event rate observed in ATTR-ACT. NYHA class is reported in clinical routine in virtually every patient with heart failure and ATTR-CM. About 90% of current patients treated with tafamidis are in NYHA class II or III and will move between these classes during treatment [4,18,19,23]. Due to the lack of objectively measurable characteristics, the assignment to NYHA class II or III is like flipping a coin and might not be a helpful marker in assessing the disease course. Progression of the laboratory biomarker domain in our cohort was driven by NT-pro-BNP levels. Natriuretic peptide levels are affected by several dynamic factors such as volume status, glomerular filtration rate or atrial fibrillation [24], which should be considered when interpreting results. However, serial changes in ECV assessed by CMR show a reasonable correlation with changes in NT-pro-BNP levels in patients with ATTR-CM suggesting that this biomarker may pragmatically be used as an resource-saving alternative to CMR [18].

Progression of the imaging/ECG domain was most common in our cohort and was driven by functional rather than morphological echocardiographic markers. This is not unexpected given the short follow-up time that may have precluded the detection of structural changes. Giblin et al. reported a similar rate of deteriorating functional parameters, GLS and E/e’ in 23 patients with tafamidis treatment (91% wild-type ATTR) at 12 months follow-up [20]. Interestingly, the high rate of 50% of progressive septal hypertrophy [22] was not seen in the ATTR-ACT trial despite longer follow-up. This may be explained by the interobserver variability of this marker in clinical routine that may have led to an overestimation of progress [25]. Importantly, all echocardiographic measures in our study were performed by one experienced physician.

CMR is the gold-standard for non-invasive quantification of cardiac amyloid load, and measures such as ECV have strong prognostic value [26], but its role in monitoring disease progression in ATTR-CM is currently unclear [5]. Assuming an ECV threshold for defining disease progress, which has been derived from light-chain amyloidosis, we observed 18% of patients with progress in our subgroup analysis of 22 patients. Comparable results have been published by Rettl and Chamling in studies with a slightly longer follow-up [18,19]. Interestingly, albeit ECV assessed by CMR is significantly correlated with the amyloid load assessed from myocardial biopsy, native T1, LV mass index and troponin T showed a stronger correlation with the amyloid load than ECV [27]. Considering factors such as costs and availability, coupled with the absence of a significant correlation between ECV and clinical domains in our cohort, additional data are necessary to define the role of CMR in monitoring patients undergoing ATTR therapy.

The primary aim of monitoring patients is to guide decisions on how to proceed with therapy. Currently, due to the lack of therapeutic alternatives in wild-type ATTR, the main clinical question is whether to continue a cost intensive therapy or to stop therapy because markers indicate futility. To answer the latter, markers under evaluation need to be correlated with clinical outcomes. Of note, a large analysis of patients with cardiac ATTR prior to approval of tafamidis showed that the only serial echocardiographic parameters associated with mortality were worsening in mitral regurgitation and deterioration of stroke volume index [28]. However, it is unclear whether these results can be transferred to patients with tafamidis therapy.

### Limitations

This study has several limitations. The analysis must be interpreted as a pilot study with limited follow-up duration (6 months), small sample size, and limited statistical power to detect significant changes in the disease course. However, all previous studies were comparably smaller-sized. Following the recent expert consensus on monitoring of ATTR patients, we report first-hand data on ATTR progression monitoring under tafamidis as well as including CMR characterization in a subgroup of our cohort. It must be noted that the parameter 6 min walk distance was not part of our standard evaluation during the study period, hence this data could not be included into the analyses.

The retrospective single center design limits extrapolation to other health care systems and settings. In order to refine the suggested multiparametric monitoring concept, individual features and domains need to be correlated with patient outcome in future analyses.

## 5. Conclusions

In conclusion, we report the first frequency estimates of markers of disease progression following a recent expert consensus statement. The most common features with demonstrable short-term progress were echocardiographic parameters of LV function like the grade of diastolic dysfunction and GLS. Disease progress assessed by CMR was less common in our subgroup analysis and showed no association with clinical domains. Our results contribute to refining the multiparametric monitoring concept in patients with ATTR-CM and also highlight the limitations of currently available ATTR-CM treatment in halting disease progression.

## Figures and Tables

**Table 1:** Baseline characteristics of ATTR-CM patients.

Variable	*n* = 62
Clinical characteristics	
Male gender, *n* (%)	55 (88.7)
Age (y), mean (SD)	79.0 ± 6.4
NYHA functional class, *n* (%)	
I	11.3
II	54.8
III	33.9
IV	-
Coronary artery disease, *n* (%)	25 (40.3)
Arterial hypertension, *n* (%)	46 (74.2)
Atrial fibrillation or flutter, *n* (%)	37 (59.7)
Previous pacemaker implantation, *n* (%)	6 (9.7)
Polyneuropathy, *n* (%)	34 (54.8)
Spinal canal stenosis, *n* (%)	7 (11.3)
Carpal tunnel syndrome, *n* (%)	28 (45.2)
Cerebrovascular disease, *n* (%)	6 (9.7)
Prior heart valve intervention, *n* (%)	7 (11.3)

Values are presented as total numbers *n* and %, mean ± standard deviation (SD) or median and interquartile range (IQR). NYHA, New York Heart Association.

**Table 2:** Functional, echocardiographic and CMR characteristics of ATTR-CM patients at baseline and 6-months follow-up.

Variable	Baseline *n* = 62	6-Mo FU *n* = 62	*p*-Value
Functional parameters			
NYHA functional class, *n* (%)			<0.01
I	11.3	12.9	
II	54.8	64.5	
III	33.9	22.6	
IV	-	-	
MLHFQ (points), median (IQR)	36 (20–49)	32 (16–45)	0.866
Laboratory parameters			
Troponin T (ng/L), median (IQR)	0.053 (0.036–0.088)	0.048 (0.032–0.077)	0.001
NT-proBNP (pg/mL), median (IQR)	2.177 (1.271–3.848)	2.125 (1.145–3.674)	0.353
eGFR (ml/min/1.73 m^2^), median (IQR)	59 (47–74)	54 (41–67)	0.018
NAC Stage			<0.01
I	35 (57.4)	34 (57.6)	
II	18 (29.5)	18 (30.5)	
III	8 (13.1)	7 (11.9)	
Imaging and ECG data			
Impaired LVEF (<50%)	11 (17.7)	11 (17.7)	1.0
Interventricular septum (mm)	17 (16–18)	17 (16–18.5)	0.317
LV stroke volume index (mL/m^2^)	30.6 (25.7–38.5)	33.6 (28.8–47.5)	<0.01
LV global longitudinal strain (%)	−11.0 (−8.5 to −14.2)	−11.5 (−8.8 to −13.8)	0.877
Diastolic dysfunction ≥ grade II	35 (57.4)	39 (62.9)	0.034
LA area (cm^2^)	45.1 (39.0−53.7)	48.2 (41.6–58.0)	0.095
AV block (of any degree)	16 (25.8)	20 (32.3)	<0.01
*CMR*	*n* = 22	*n* = 22	
T1 relaxation time (ms)	1095 (1026–1119)	1074 (1045–1100)	0.758
ECV (%)	46 (41–51)	47 (43–55)	0.082

Values are presented as total numbers n and %, mean ± standard deviation (SD) or median and interquartile range (IQR). NYHA, New York Heart Association; MLHFQ, Minnesota Living with Heart Failure Questionnaire; eGFR, estimated glomerular filtration rate; NAC, national amyloidosis center; ECG, electrocardiogram; LVEF, left ventricular ejection fraction; LV, left ventricle; LA, left atrium, AV, atrioventricular; CMR, cardiac magnetic resonance; and ECV, extracellular volume.

## Data Availability

Data cannot be made publicly available for ethical reasons.
